# Analysis of Connectome Graphs Based on Boundary Scale

**DOI:** 10.3390/s23208607

**Published:** 2023-10-20

**Authors:** María José Moron-Fernández, Ludovica Maria Amedeo, Alberto Monterroso Muñoz, Helena Molina-Abril, Fernando Díaz-del-Río, Fabiano Bini, Franco Marinozzi, Pedro Real

**Affiliations:** 1 Higher Technical School of Informatics Engineering, University of Seville, Avda. Reina Mercedes, s/n, 41012 Seville, Spain; mjmoron@us.es (M.J.M.-F.); albertomonterrosomunoz@gmail.com (A.M.M.); habril@us.es (H.M.-A.); fdiaz@us.es (F.D.-d.-R.); real@us.es (P.R.); 2 Department of Mechanical and Aerospace Engineering, “Sapienza” University of Rome, Via Eudossiana, 18, 00184 Rome, Italy; fabiano.bini@uniroma1.it (F.B.); franco.marinozzi@uniroma1.it (F.M.)

**Keywords:** connectome, hypergraph theory, Betti numbers, topological scale

## Abstract

The purpose of this work is to advance in the computational study of connectome graphs from a topological point of view. Specifically, starting from a sequence of hypergraphs associated to a brain graph (obtained using the Boundary Scale model, $BS^{2}$), we analyze the resulting scale-space representation using classical topological features, such as Betti numbers and average node and edge degrees. In this way, the topological information that can be extracted from the original graph is substantially enriched, thus providing an insightful description of the graph from a clinical perspective. To assess the qualitative and quantitative topological information gain of the $BS^{2}$ model, we carried out an empirical analysis of neuroimaging data using a dataset that contains the connectomes of 96 healthy subjects, 52 women and 44 men, generated from MRI scans in the Human Connectome Project. The results obtained shed light on the differences between these two classes of subjects in terms of neural connectivity.

## 1. Introduction

The study of brain graphs [1], which represent the brain’s functional and structural connections through a network of nodes and edges, is of paramount importance to understanding the functioning of the human brain and studying potential differences between individuals. Moreover, the study of brain graphs has helped, in the past, and can currently help to identify potential bio-markers for various neurological disorders [2,3]. In the last years, several scientific research studies have been published that develop and propose different methods for describing, studying, and analyzing brain networks, which are referred to as “connectomes” [4]. Among the most widely used methods for studying brain graphs, we certainly find those based on the use of graph theory metrics [5,6] and on persistent homology [7,8]. Some studies have analyzed datasets containing only information from healthy individuals [9,10], while others have used the developed tools and cited methods to analyze and compare brain graphs from both healthy and pathological subjects [11,12,13].

**Figure 1:**
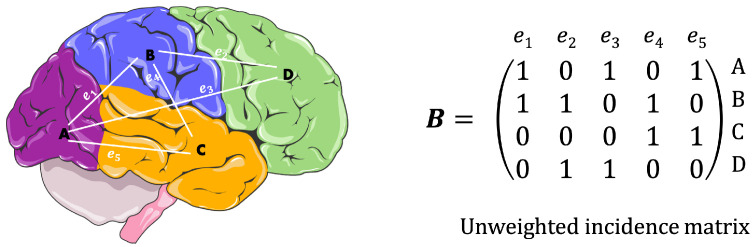
Example of a simple brain graph and its associated incidence matrix. Figure modified with text, markings, and annotation after adaptation of "Brain” from Servier Medical Art by Servier, licensed under a Creative Commons Attribution 3.0 Unported Licence. Original photo adapted from https://smart.servier.com/smart_image/brain-area/, accessed on 17 May 2023.

In this work, an innovative software tool developed in Python language is presented for the analysis of brain graphs, based on the new “Topological Scale Framework” (TSF) [14,15]. More concretely, the set of algorithms proposed here conforms an iterative process that uses as initial value the incidence matrix (Figure 1) of the original brain graph, to gradually generate a sequence of associated hypergraphs parameterized by a scale of topological nature. This sequence is also called boundary-scale model of the brain graph. A set of local and global topological numerical indices (such as lists of nodes and edges degrees and Betti numbers) are collected for each hypergraph component of the model. This tool also includes some of the most commonly used graph metrics in network neuroscience [16] in order to compare it with other well-known analysis methods.

The database on which the boundary-scale model has been tested consists of 96 undirected and unweighted brain graphs of healthy subjects, 44 males and 52 females, generated from MRI scans obtained from the Human Connectome Project (HCP) [17]. The developed tool is then used to evaluate possible sex differences in brain connectivity, adhering to previous studies [18,19,20,21] and opening up new research perspectives in this field.

## 2. Materials and Methods

This section explains the mathematical background and notation strictly necessary to understand the nature of the topological space-scale method and describes the dataset used for the present study.

Let $n_{1}$ and $n_{2}$ be two natural numbers with $n_{1}\leq n_{2}$. The interval $\left[n_{1},n_{2}\right]$ means the set $\left\{n\in N\;|\;n_{1}\leq n\leq n_{2}\right\}$. An enumeration function $\ell_{A}:\left[1,n\right]\to A$ (for some n∈N) for a finite set *A* is a bijection. The cardinal of *A* is |A|=n. Let us denote by ∗ the product of matrices with values in the field $Z_{2}=\left\{0,1\right\}$. If *B* is a matrix of *m* files and *n* columns (m,n∈N), $B^{T}$ denotes the transpose of *B* of *n* files and *m* columns. If m=n, $B^{k}=\underset{k\;\mathrm{times}}{\underset{︸}{B*B*\cdots *B}}$ (k∈N) and $B^{0}=I_{m,m}$, being $I_{m,m}$ the identity matrix of dimensions m×m.

### 2.1. Fundamentals of Hypergraph Theory

**Figure 2:**
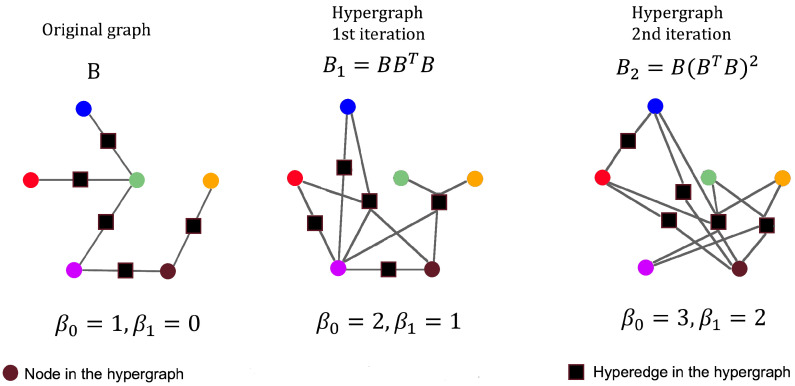
First hypergraphs’ components of the $BS^{2}$-model of a six-vertex graph and their corresponding Betti numbers.

An (incidence) *hypergraph* is a tuple $G=(\left(V,\ell_{V}\right),\left(E,\ell_{E}\right),I)$, where $\left(V,\ell_{V}\right)$ and $\left(E,\ell_{E}\right)$ are enumerated finite sets called *vertices* and *edges*, respectively, and I⊂V×E is the *vertex-edge incidence relation* of *G*. Let us note that a node-edge incidence relation *I* can also be identified with a *vertex-edge incidence matrix* $B\left(G\right)=\left[b_{i,j}\right]$ of dimension |V|×|E| with $b_{i,j}=1$ if $\ell^{V}\left(i\right)=v$; $\ell^{E}\left(j\right)=e$ and (v,e)∈I(G), and zero otherwise, for i∈[1,|V|], j∈[1,|E|]. From now on, we omit the enumeration functions on vertices and edges and any hypergraph *G* is defined by the three-tuple (V,E,B(G)). Note that this definition differs from the classical one where each edge of a hypergraph is identified with a finite subset of *V* (Figure 2).

Two kinds of topological features or indices can be distinguished: local and global.

As an example of local topological features, we have the *degree* dgr(w,G) of a vertex (resp. an edge) *w*, that is, the number of edges (resp. vertices) which are related to the vertex (resp. the edge). Both features can be derived from the incidence matrix of the hypergraph, as the sum of 1 s for each column to obtain the vertex degree and the sum of 1 s for each row to obtain the edge degree. The edge degree index allows us to define the notion of graph. *An (incidence) graph* G=(V,E,B) is a hypergraph with every edge having degree two.

Relevant global topological indices of a hypergraph G=(V,E,B) are the Euler and Betti numbers. The Euler number of a hypergraph *G* is the integer number χ(G)=|V|∖|E|. The *k*-th Betti number $\beta_{k}\left(G\right)$ (k=0,1) of *G* is the dimension of the *k*-th homology vector space of *G* with coefficients in $Z_{2}$. Both $\beta_{0}\left(G\right)$ and $\beta_{1}\left(G\right)$ are classically computed using the Smith normal form of the incidence matrix *B* of *G* [22]. The Euler number of a hypergraph *G* is strongly related to its Betti numbers via this formula: $\chi \left(G\right)=\beta_{0}\left(G\right)\setminus \beta_{1}\left(G\right)$. Note that, given a graph, Betti numbers have an intuitive interpretation. In this case, $\beta_{0}$ corresponds to the number of connected components and $\beta_{1}$ to the number of elementary cycles of the graph.

### 2.2. Boundary-Scale Theory for Hypergraphs

The fundamental limitation of graphs is that merely pairwise interactions are captured, whereas many real and biological systems exhibit group interactions. In fact, as the authors of [23] recognize, simplicial complexes and hypergraphs are natural candidates for describing higher order interactions. The *boundary scale model* ($BS^{2}$-model, for short) provides a tool to transform a graph into a sequence of hypergraphs as a generalization of the former pairwise interactions, allowing for the exploration of multiple and complex relations in higher dimensions.

The $BS^{2}$-model of a hypergraph G=(V,E,B) is a sequence of hypergraphs ${\mathrm{BS}}^{2}\left(G\right)={\left({\mathrm{BS}}_{s}^{2}\left(G\right)\right)}_{s\geq 1}$, being ${\mathrm{BS}}_{s}^{2}\left(G\right)=\left(V,E,B_{s}\right)$ the hypergraph at scale *s*, with $B_{s}={\left(B*B^{T}\right)}^{s-1}*B$, s≥1 (see Table 1). Moreover, this highly redundant representation of *G* involves transition maps $\rho_{s}:{\mathrm{BS}}_{s}^{2}\left(G\right)\to {\mathrm{BS}}_{s-1}^{2}\left(G\right)$, defined by $\rho_{s}\left(v\right)=v$∀v∈V and $\rho_{s}\left(e\right)=B^{T}*B*e$∀e∈E, ∀s>1. Note that *e* is considered here as a vector. They connect consecutive hypergraph components, preserving homological information [14,15]. Extracting from a $BS^{2}$-model classical and new (local and global) topological indices is the method of TSF for topologically discriminating brain graphs.

An example graphically showing the first three-component hypergraphs (s=1,2,3) of $BS^{2}$-model for a simple six-vertex graph is given in Figure 2. Note that the number of vertices and edges remains unaltered throughout the levels of the model. Only incidence connections among them are being modified. Vertices are shown using circles of different colors and they have the same spatial distribution for all the boundary scale indices. Edges are indistinctly described by black solid squares. For simplicity’s sake, transition maps are omitted. Betti numbers ($\beta_{0}$ and $\beta_{1}$) are presented for each hypergraph of the $BS^{2}$-model in Figure 2.

### 2.3. Topological Brain Network Analysis

We work here with intra-analysis metrics of the $BS^{2}$-model. That means that, given a local (resp. global) topological index ind(w,G) (resp. simply ind(G)) of a hypergraph *G*, we focus on the sequence ${\left(ind\left(w,G_{s}\right)\right)}_{s\geq 1}$ (resp. ${\left(ind\left(G_{s}\right)\right)}_{s\geq 1}$). Inter-analysis of the $BS^{2}$-model is based on sequences in which transition functions of the model are involved. The extended study of brain graphs adding inter-analysis metrics is intended to be in the near future.

At each topological scale *s*, we deal here with the local index $deg(w,G_{s})$ and the global features χ(G), $\beta_{0}\left(G_{s}\right)$ and $\beta_{1}\left(G_{s}\right)$. These parameters are obtained from the incidence matrix of every hypergraph component.

**Figure 3:**
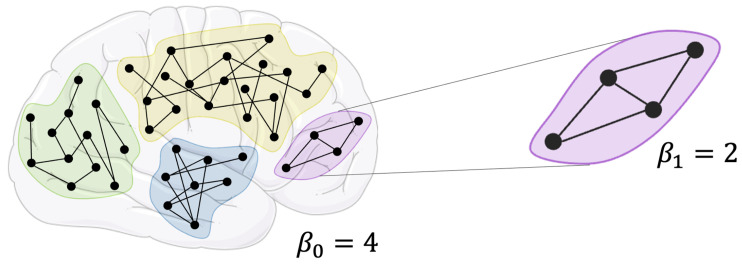
Example of Betti numbers $\beta_{0}$ and $\beta_{1}$ for a graph. Figure modified with markings and annotation after adaptation of “Brain” from Servier Medical Art by Servier, licensed under a Creative Commons Attribution 3.0 Unported Licence. Original photo adapted from https://smart.servier.com/smart_image/brain/, accessed on 17 May 2023.

The Betti numbers $\beta_{0}$ and $\beta_{1}$ determine the number of homological holes of dimension 0 and 1 of the given hypergraph. In the case in which *G* is a graph, $\beta_{0}$ coincides with the number of path-connected components of *G* and $\beta_{1}$ identifies the number of independent loops or circuits within each path-connected component (Figure 3).

### 2.4. Dataset

The data source of this study is the website of the Human Connectome Project at the address www.humanconnectome.org, accessed on 17 May 2023 [17]. The National Institutes of Health–funded large Human Connectome Project (HCP) regularly provides its high-angular resolution diffusion imaging (HARDI) Magnetic Resonance Imaging datasets of hundreds of healthy human subjects. State-of-the-art computational methods have made possible the identification of 1015 gray matter areas of the brain (ROI, Region Of Interest) and the connections between them [24]. Starting from the HARDI, brain graphs can be obtained: each one of the 1015 ROI sets can correspond to a node (or a vertex) and the edges of the graphs can be labeled by physical properties of the neural fibers connecting the corresponding ROIs. Once one brain graph for each subject is obtained, since the nodes of these graphs correspond to the very same set of 1015 anatomical areas, one can make comparisons between the brain graphs of individual subjects or groups of subjects in several ways [25,26]. The brain graphs, analyzed in the present work, can be downloaded at the site braingraph.org, accessed on 17 May 2023, selecting “Partial set, 96 brains, 20,000 streamlines”. For this study, the data were downloaded in December 2022.

The dataset [27] contains the connectomes of 96 healthy subjects, 52 females and 44 males, between the ages 22 and 35, each with 83, 129, 234, 463, and 1015 node resolution. Each graph is available as a separate GraphML file with a standardized name: nnnnnn_connectome_scale_xxx.graphML. The first six digits (nnnnnn) refer to the subject’s ID from the HCP’s public release; and the last digits (xxx), which can be 2 or 3, refer to the number of vertices in the graph. Scale 33 corresponds to 83 vertices, scale 60–129 vertices, scale 125–234 vertices, scale 251–463 vertices, and scale 500–1015 vertices [27]. A group of undirected and unweighted brain graphs have been selected from this dataset.

### 2.5. Statistical Analysis

The statistical null hypothesis tested is that the graph parameters do not differ between the male and female groups. The first approach was to apply ANOVA [28]; therefore, as the first step, we checked the assumption of the said statistical test. These are homogeneity of variance and normal distribution of data, respectively tested with the Levene’s test [29] and the Kolmogorov-Smirnov test [30]. When the results of both tests were satisfactory, ANOVA was applied. Instead, where one or both tests did not lead to a positive result, a different statistical test was chosen to analyze the data. In particular, data that did not conform to the assumption of homogeneity of variance were analyzed using Welch’s alternative to ANOVA [31]; data that did not conform to the normality test were subjected to a non-parametric test, specifically the Mann–Whitney U test, which is also known as the Wilcoxon rank sum test [32,33]. In those cases analyzed where the *p*-value was less than 0.05, it was possible to reject the null hypothesis, meaning that all the corresponding brain graph parameters differ significantly in sex groups at a significance level of 5%. We used MATLAB (2022a) for the statistical analysis.

## 3. Results

The methodologies described in the previous section have yielded results that will be reported here.

**Figure 4:**
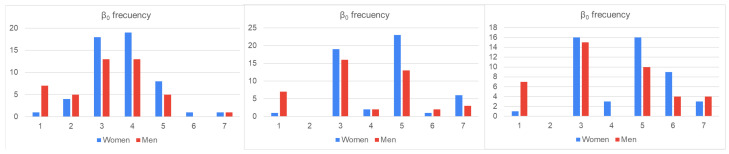
0-Betti number bar charts computed for the first 3 iterations of the model.

**Figure 5:**
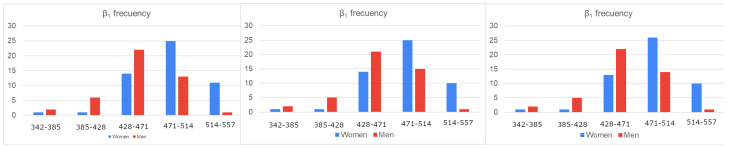
1-Betti number bar charts computed for the first 3 iterations of the model.

Before addressing the statistical analysis, the gain in local and global topological information extracted from the $BS^{2}$ model with regard to that directly obtained from the original connectome graph can be easily visualized. Bar charts for Betti numbers of dimensions 0 and 1 of the first three hypergraph components of the $BS^{2}$ model of connectome graphs are displayed in Figure 4 and Figure 5. The *X*-axis of these charts denote the different integer values of the parameters $\beta_{0}$ and $\beta_{1}$ and the *Y*-axis measures the number of individual having the same Betti number. Charts of women’s frequencies are colored blue, and men’s frequencies are colored red. Note that the left charts correspond to the original graph database.

Now, the results of the statistical analysis for each feature and the corresponding statistical test applied have been reported in Table 2, where:

- node_deg_xxx is the average degree of the nodes, xxx can assume different values: or if it is referred to the original graph; 1it, 2it, 3it if it is referred to the first, second, third iteration, respectively.
- edge_deg_xxx is the average degree of the edges, xxx can assume different values: 1it, 2it, 3it if it is referred to the first, second, third iteration, respectively.
- beta0_xxx is the Betti number $\beta_{0}$ of the graph at the first, second, and third iterations.
- beta1_xxx is the Betti number $\beta_{1}$ of the graph at the first, second, and third iterations.

**Table 2:** Results of the statistical analysis of the different parameters (average degree of nodes and edges, $\beta_{0}$ and $\beta_{1}$) computed for the 83-vertex, 129-vertex, 234-vertex, 463-vertex, and 1015-vertex brain graphs.

Feature	Statistical Test Applied	*p*-Value
83-nodes resolution		
node_deg_or	ANOVA	**0.00027118**
node_deg_1it	ANOVA	**0.00017121**
node_deg_2it	ANOVA	**0.00046663**
node_deg_3it	ANOVA	**0.00025789**
edge_deg_1it	WANOVA	**0.0021**
edge_deg_2it	ANOVA	**0.0181**
edge_deg_3it	WANOVA	**0.0218**
beta0_1it	MW-U test	0.0677
beta0_2it	MW-U test	0.1124
beta0_3it	MW-U test	0.1801
beta1_1it	ANOVA	**0.00012318**
beta1_2it	ANOVA	**0.00021162**
beta1_3it	ANOVA	**0.0001278**
129-nodes resolution		
node_deg_or	ANOVA	**0.0211**
node_deg_1it	ANOVA	**0.0001994**
node_deg_2it	ANOVA	**0.00043659**
node_deg_3it	ANOVA	**0.00049557**
edge_deg_1it	ANOVA	**0.00007947**
edge_deg_2it	ANOVA	**0.0011**
edge_deg_3it	ANOVA	**0.0024**
beta0_1it	MW-U test	0.5185
beta0_2it	MW-U test	0.7152
beta0_3it	MW-U test	0.9758
beta1_1it	ANOVA	**0.002**
beta1_2it	ANOVA	**0.0031**
beta1_3it	ANOVA	**0.0021**
234-nodes resolution		
node_deg_or	MW-U test	**0.0005184**
node_deg_1it	ANOVA	**0.00023928**
node_deg_2it	ANOVA	**0.00022495**
node_deg_3it	ANOVA	**0.00058833**
edge_deg_1it	ANOVA	**0.0000049857**
edge_deg_2it	ANOVA	**0.000010999**
edge_deg_3it	ANOVA	**0.000077484**
beta0_1it	MW-U test	0.7544
beta0_2it	MW-U test	0.8749
beta0_3it	ANOVA	0.8761
beta1_1it	MW-U test	**0.00041007**
beta1_2it	MW-U test	**0.00054039**
beta1_3it	MW-U test	**0.00039878**
463-nodes resolution		
node_deg_or	ANOVA	0.3238
node_deg_1it	MW-U test	**0.000050071**
node_deg_2it	ANOVA	**0.0014**
node_deg_3it	ANOVA	**0.0018**
edge_deg_1it	ANOVA	**0.000010282**
edge_deg_2it	ANOVA	**0.000004063**
edge_deg_3it	ANOVA	**0.0000054781**
beta0_1it	ANOVA	0.4886
beta0_2it	ANOVA	0.558
beta0_3it	ANOVA	0.5718
beta1_1it	ANOVA	0.2746
beta1_2it	ANOVA	0.2781
beta1_3it	ANOVA	0.2762
1015-nodes resolution		
node_deg_or	ANOVA	0.5788
node_deg_1it	MW-U test	**0.0189**
node_deg_2it	ANOVA	**0.006**
node_deg_3it	ANOVA	**0.0092**
edge_deg_1it	ANOVA	**0.000125**
edge_deg_2it	ANOVA	**0.000015016**
edge_deg_3it	ANOVA	**0.000047532**
beta0_1it	ANOVA	0.1716
beta0_2it	ANOVA	0.1616
beta0_3it	ANOVA	0.1644
beta1_1it	MW-U test	0.4186
beta1_2it	MW-U test	0.425
beta1_3it	MW-U test	0.4271

In addition, those *p*-values that were individually less than the threshold have been highlighted in bold.

Among all the computed and analyzed parameters, the Average Nodes Degree (AND) and the Average Hyperedges Degree (AHD) successfully pass the statistical test for all five analyzed nodal resolutions. The $\beta_{0}$, which identifies the number of connected components, does not pass the statistical tests for any nodal resolution. On the other hand, the $\beta_{1}$ passes the statistical tests for lower resolutions (83, 129, 234 nodes), but for higher resolutions, starting from 463 nodes, the associated *p*-value begins to be greater than 0.05, meaning that the parameter does not pass the test.

**Figure 6:**
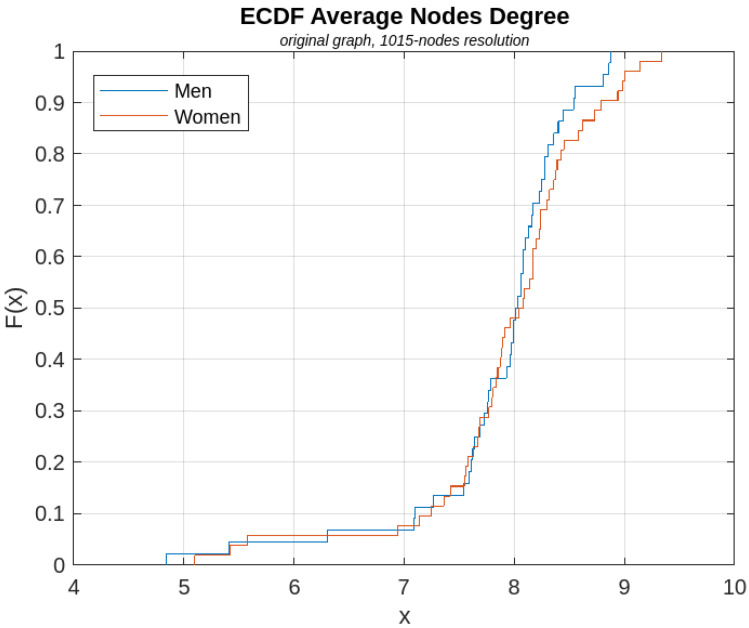
ECDF of the AND parameter computed for the original graph.

**Figure 7:**
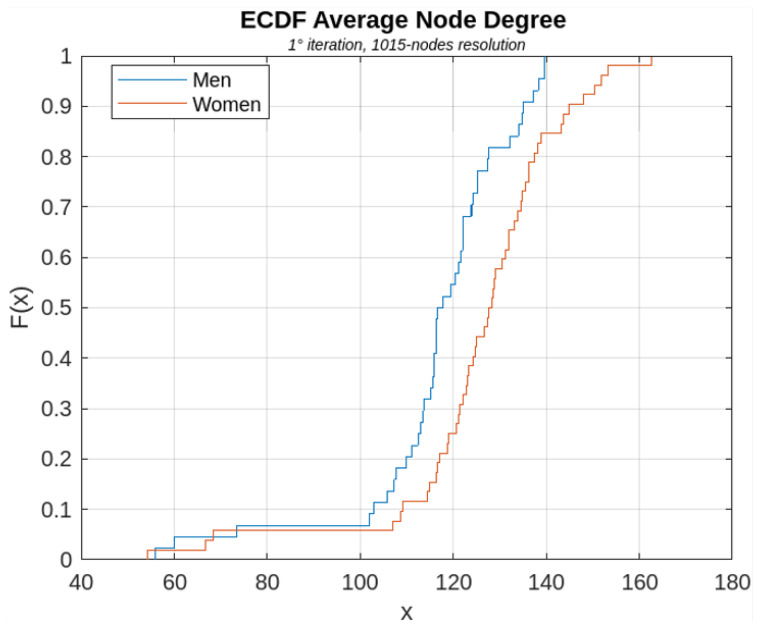
ECDF of the AND parameter computed for the first iterations of the $BS^{2}$ iterative process.

**Figure 8:**
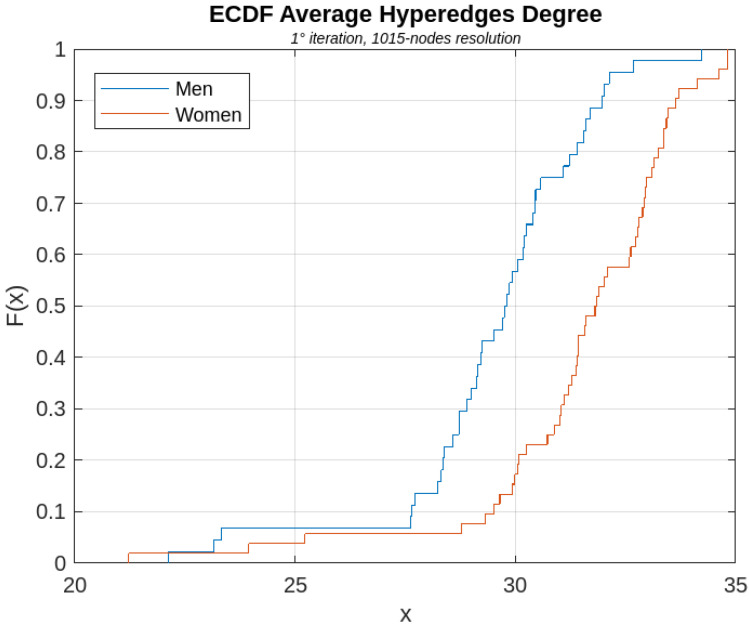
ECDF of the AHD parameter computed for the first iteration of the $BS^{2}$ iterative process.

**Figure 9:**
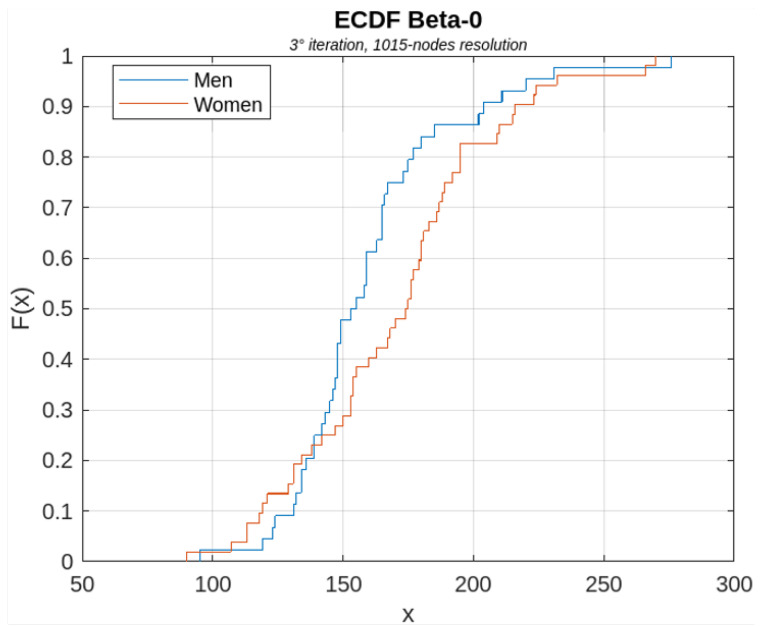
ECDF of the $\beta_{0}$ parameter computed for the first iteration of the $BS^{2}$ iterative process.

**Figure 10:**
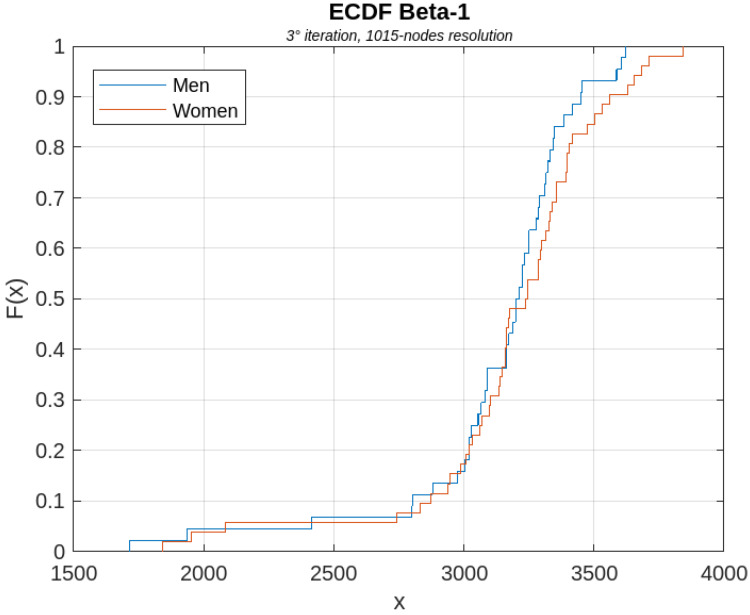
ECDF of the $\beta_{1}$ parameter computed for the first iteration of the $BS^{2}$ iterative process.

Figure 6, Figure 7, Figure 8, Figure 9 and Figure 10 show, for illustrative purposes, the empirical cumulative distribution functions (ECDFs) [34] related to the four parameters studied, all of them computed for the highest nodal resolution, that is, 1015 nodes.

In Figure 6, Figure 7, Figure 8, Figure 9 and Figure 10, the parameter value (*x*) is plotted on the x-axis, and the fraction of subjects F(x) which has the value of that parameter at most *x*, is plotted on the y-axis. In other terms, for each value of *x*, on the horizontal axis, the curves demonstrate the male (blue curve) and female (red curve) fraction of subjects with the value of the parameter under analysis at most *x*. For example, with reference to Figure 8, for x=30, 16% of the females have the Average Node Degree value less than *x*, while about 57% of the males have the same value less than *x*.

## 4. Discussion

Among all the computed parameters, in particular, the AND and the AHD (with reference to Figure 7 and Figure 8) were found to be significantly different in statistical terms between women and men, for all iterations of the $BS^{2}$ process and for all analyzed nodal resolutions. Specifically, both parameters turned out to be higher in female connectomes rather than in male ones, leading to the conclusion that female brain graphs are more connected than the connectome of males.

The Average Nodes Degree parameter computed for high nodal resolution is of particular interest and demands focused consideration because, while the statistical difference between sexes was not significant in the evaluation of the original graph, it was found to be statistically significant when analyzed for the different iterations of the $BS^{2}$ process. This result is of particular importance because it highlights the potential power and relevance of applying this theory to brain graphs.

However, other considered parameters, such as the $\beta_{0}$ and the $\beta_{1}$, from the conducted analysis have not been found to be characteristic of the two sexes; the first one for all nodal resolutions analyzed and the second one only for high nodal resolutions. In fact, since they did not pass the statistical tests, we cannot conclude, from this preliminary application of the tool, that they are an efficient and significant indicator of the difference between the female and male sexes in terms of brain connectivity. This may have arisen from the fact that the brain graphs of women and men do not actually differ in terms of the number of connected components and the number of cycles, or from the limited number of graphs examined in the present analysis.

The analysis conducted has provided new parameters such as the Average Nodes Degree, and the Average Hyperedges Degree, which have turned out to be metrics that highlight the difference in brain connectivity between the two sexes. On the other hand, the $\beta_{0}$ and the $\beta_{1}$ have been found to be insensitive to the sex difference in terms of brain connectivity.

Finally, the authors would like to emphasize that the aim of the work presented is to extend the information that can be analyzed for a given network through the application of the $BS^{2}$ model. We acknowledge that, if the present study had a clinical objective, it would have been imperative to expand both the dataset and the number of iterations for the $BS^{2}$ model.

## 5. Conclusions

The work was carried out with the aim of applying a Boundary Scale-Space model to brain graphs. A preliminary application of the proposed tool has been conducted on the described database, studying the differences in terms of neural connectivity between the two sexes, adhering to previous scientific studies that have carried out a similar analysis [18,19,20]. Indeed, this initial application of the tool was carried out on healthy subjects with the aim of identifying a group of subjects that could serve as a control group in future studies where differences, not based on sex as conducted in this study, will be analyzed between pathological and physiological subjects or between brain graphs of the same patient but at different stages of the disease.

The results presented here demonstrate that the use of the boundary scale model for the analysis of brain graphs has led to a significant expansion of the results of Szalkai B. et al. (2015) [21]. Note that only a small part of the information that can be obtained through the $BS^{2}$ sequence has been employed in this work. We are convinced that the future development of improved scale-space topological methods to quantify the topology of brain networks will provide models capable of describing basic interactions between neuronal ensembles and to predict network topological alterations correlated to cognitive/motor behavior and disease. In fact, due to the generic nature of the mathematical software associated to TSF representations, its impact could be significant in other areas of Biomedical Data Science, like Radiomics Analysis [35] or Knowledge Graphs [36].

## Figures and Tables

**Table 1:** $BS^{2}$ Intra-analysis Process.

	${\mathrm{bs}}^{2^{1}}$	${\mathrm{bs}}^{2^{2}}$	${\mathrm{bs}}^{2^{3}}$	⋯	${\mathrm{bs}}^{2^{i}}$
**Incidence matrix**	*B*	$BB^{T}B$	$B{\left(B^{T}B\right)}^{2}$	⋯	$B{\left(B^{T}B\right)}^{i-1}$
**Global features**	$\beta_{0}^{1}$	$\beta_{0}^{2}$	$\beta_{0}^{3}$	⋯	$\beta_{0}^{i}$
	$\beta_{1}^{1}$	$\beta_{1}^{2}$	$\beta_{1}^{3}$	⋯	$\beta_{1}^{i}$
	$\chi^{1}$	$\chi^{2}$	$\chi^{3}$	⋯	$\chi^{i}$
**Local features**	$n-deg^{1}$	$n-deg^{2}$	$n-deg^{3}$	⋯	$n-deg^{i}$
	$h-deg^{1}$	$h-deg^{2}$	$h-deg^{3}$	⋯	$h-deg^{i}$

## Data Availability

The results presented in this study are available from the author M.J.M.-F. on request.

## Abbreviations

The following abbreviations are used in this manuscript:

MRI	Magnetic Resonance Imaging
TSF	Topological Scale Framework
HCP	Human Connectome Project
HARDI	High-Angular Resolution Diffusion Imaging
ROI	Region of Interest
AND	Average Nodes Degree
AHD	Average Hyperedges Degree
ECDF	Empirical Cumulative Distribution Function
$BS^{2}$	Boundary Scale Space
